# Wireless Chipless System for Humidity Sensing

**DOI:** 10.3390/s18072275

**Published:** 2018-07-13

**Authors:** José F. Salmerón, Andreas Albrecht, Silmi Kaffah, Markus Becherer, Paolo Lugli, Almudena Rivadeneyra

**Affiliations:** 1 Institute for Nanoelectronics, Technical University of Munich, 80333 München, Germany; jf.salmeron@tum.de (J.F.S.); andreas.albrech@tum.de (A.A.); silmi.kaffah@tum.de (S.K.); markus.becherer@tum.de (M.B.); 2 Faculty of Science, Free University of Bolzen-Bolzano, 39100 Bolzano, Italy; paolo.lugli@tum.de

**Keywords:** flexible substrate, inductive coupling, inkjet printing, printed electronics, reader, resonance frequency

## Abstract

This work describes a fully wireless sensory system where a chipless strategy is followed in the sensor part. Alternatively, to characterize only the sensing element, we present the response of the reader antenna when the sensing element is placed in its vicinity: changes in the parameter of interest are seen by the reader through inductive coupling, varying its frequency response. The sensing part consists of a LC circuit manufactured by printing techniques on a flexible substrate, whose electrical permittivity shows dependence with the moisture content. The measurement distance show significant differences in the frequency response: a change of 700 kHz is observed when the measurement is performed directly on the wireless chipless sensor between 20% and 80%RH, while this variation in frequency is reduced more than three times when measuring at the reader antenna with 5 mm distance between elements. Furthermore, we demonstrate the importance of the separation between reader and sensor to get a reliable measuring system.

## 1. Introduction

Wireless sensors are a recent trend in sensor design based on remote access to the desired information via wireless link, making monitoring under harsh conditions or in hazardous environments easier. Typical wireless sensors can be classified into surface acoustic wave sensors, intermodulation sensors, optical sensors, and radiofrequency (RF) sensors. Among RF sensors, it can be differentiated between electromagnetically coupled sensors and devices working on the far or near field regions, depending on the operating frequency. In the latter case, the coupling mechanism is inductive normally using LC resonant circuits [1,2,3,4,5,6,7,8,9]. This work is centered in LC-type passive sensors that are simple structures, easy to integrate, and cheap to produce [10]. In addition, this kind of sensor is highly energy efficient because of its low operating frequency and its smaller coupling distance [11,12,13]. Because of all these positive features, there have been many efforts in the last years to develop LC sensors for different applications, like pH control [14], temperature [11,13], humidity [9], and pressure in the human body [15]. The sensing system consists of an LC circuit, which is powered and interrogated remotely by a reader based on a coil antenna. Variations in the chemical or physical environmental conditions lead to changes in electrical properties, such as its conductance or inductance, leading to a variation in the near field coupling properties. In order to obtain the resonance frequency wirelessly, an extra coil at the readout circuit is inductively coupled to the inductor of the LC circuit. The resulting shift in frequency is detected by the variation in the impedance of the reader coil [14,16,17]. As a consequence, parameters of interest are remotely monitored and the sensing system in a harsh environment is passively powered. For that reason, the sensing system occupies a small volume, operates at high-temperature, and offers a long service life, compared to systems with physical access by wiring and dependence on battery power [9,14,16]. Some authors have already studied the sensing capabilities of this kind of system [17,18]. They demonstrated that in order to develop a wireless sensor system based on a coupled LC resonator with low sensitivity to noise and a high range is essential to minimize the losses of the sensor. They also presented how to calculate the accuracy and the maximum distance for any wireless sensory system.

Furthermore, the fabrication techniques utilized for their manufacturing are a critical factor because they define the performance and the cost of the sensor. In this sense, one of added values to this kind of sensors is to be manufactured by printed electronics (PE). This emerging technology can produce thin, conformal, lightweight, environmentally friendly, and ultra-cost-effective structures [19]. The combination of wireless chipless sensors with PE techniques is a promising technology to build sensory systems with all the mentioned characteristics and low-cost processes. In a previous work, we presented printed LC structures on a plastic thin-film for detection of moisture content. The structures consist of a screen-printed spiral inductor working as antenna and an array of inkjet-printed interdigitated electrodes as the capacitive element, forming together the LC resonators. The sensitive layer is directly the selected substrate [20]. In the same direction, Wang et al. [21] reported a wireless humidity sensor fabricated by dry-phase patterning and screen-printing. The sensor device was composed by a planar antenna, a tuning capacitor, and a printed sensor-capacitor. The changes in humidity were measured as a shift of the resonant frequency. The sensor was acting as a resistive-type element between 150 kHz and 250 kHz with a frequency shift of about 80 kHz in the range from 10%RH to 90%RH.

Here, we have analysed the real operation conditions of printed LC circuits sensitive to humidity when they are used as a sensor node in a wireless sensor system. For this reason, the response of the wireless sensor has been studied from the reader side because this element will be in charge of collecting all the sensor information in any real scenario. In any wireless scenario, the influence of the distance between the sensing element and the reader is a crucial factor. Therefore, we have studied the response to the moisture content placing the reader at different distances of the chipless sensor tag. Finally, the effect of temperature in the system has been also analyzed as one of the most common interfering factors while measuring environmental humidity.

## 2. Materials and Methods

### 2.1. Design and Working Principle

Our purpose is to design wireless and chipless sensors readable by smart devices. To achieve that, we utilize the common frequencies and technologies already in use (i.e., Bluetooth or Wi-Fi). In particular, we look at the high frequency (HF) band (centred at 13.56 MHz) and implemented by the near field communication (NFC) and other radio frequency identification (RFID) protocols. In this frequency band, the technology working principle is based on the coupling of two coils: one acting as the reader (i.e., the coil inductor integrated in many smartphones to support NFC communication) and the other one acting as a tag-element that sends some requested information to the reader. It has been already demonstrated that we can only extract two independent parameters of the sensor circuit from a wireless measurement (i.e., resonance frequency and quality factor) [17].

Conventional RFID tags contain a silicon chip, which establishes communication with the reader and sends to it the requested information. The input impedance of such chips is capacitive (in the order of hundreds of pF). Thus, the impedance of the equivalent circuit consists of a capacitive (C) component (silicon chip) and the inductive (L) element (fabricated coil antenna). Contrary to this, in our approach there is no chip included in the tag, making it chipless. Instead, the capacitive part is defined by the designed capacitive sensor. In particular, the electrical permittivity of the selected substrate changes with the moisture content. Therefore, variations in RH produce changes in the capacitive value, inducing a shift in the resonant frequency of the chipless tag. The feasibility of this approach to sense RH was already in a previous work [20].

The system can be basically modelled as an air-core transformer. The measured impedance on the reader side can be can be expressed as

(1) $$Z_{Measured}=Z_{Reader}+\frac{4{\left(\pi fM\right)}^{2}}{Z_{Sensor}+j2\pi fL_{sensor}}$$

where *Z_Measured_* is the impedance seen from the reader side, *M* is mutual inductance, *Z_Sensor_* is the impedance sensor tag, and *L_Sensor_* is the sensor tag inductance. When there is no tag on the vicinity of the reader (*M* = 0), the measured impedance is the reader one. If one sensor tag is close enough to be magnetically coupled to the reader, the impedance, and therefore, its SRF will change. From this equation, we can estimate how sensor tag impedance influences the measured impedance on the reader side. It must be highlighted that this change depends on the *M* and therefore the distance and relative angle [22,23].

The dependency of the SFR with the distance can be explained by the decay of the magnetic coupling with distance. The figure of merit typically employed to study the magnetic coupling is the mutual inductance *M* or the coupling coefficient *k*, that are related by $k=\frac{M}{\sqrt{L_{reader}L_{sensor}}}$. The coupling factor *k* ranges from 0 (no coupling at all) to 1 (all the magnetic flux generated by the reader inductor pass through the sensor inductor).

The elements of the described system (reader and wireless LC sensor) have been designed to resonate at HF band with ADS software (Keysight EEsof EDA, Santa Rosa, CA, USA) [24]. The antenna has been designed in order that its quality factor is optimal because it offers a longer read range and a better reading of the sensor information [25,26].

### 2.2. Materials and Fabrication Process

The selected materials were DGP-40LT-15C ink (ANP, Sejong, Korea) with a solid content of 35% of silver nanoparticles dispersed in TGME (triethylene glycol monoethyl ether) for ink-jetted patterns and silver conductive paste (Sigma Aldrich, St. Louis, MO, USA) with a solid content higher than 75% for the screen printed ones. The chosen substrate was a polyimide (Kapton^®^ HN, Dupont™, Wilmington, DE, USA) with a thickness of 75 µm whose electrical permittivity changes with the moisture content as already demonstrated in [20,27]. In order to achieve a compromise in the tag performance, the capacitive array was defined by inkjet printing to reduce the distance between consecutive fingers and, therefore, to increase the sensitivity of the sensor without occupying more area whereas the coil inductor was screen printed because of its better performance with respect to the ink-jetted antenna [24]. The array of capacitance consists of 12 replicas of interdigitated electrodes (IDE) structures with a finger width of 100 µm, spacing among consecutives fingers of 100 µm, the finger length is 2 mm and 30 fingers per electrode. Then, the capacitive structures were printed with a DMP-2831™ Dimatix printer (Fujifilm Dimatix Inc., Santa Clara, CA, USA) by only one printing layer, fixing the temperature substrate to 60 °C. Finally, the patterns were dried at 120 °C for 1 h. 

Both antenna designs, reader, and sensor tag, are the same. The reader inductor consists of seven turns, with a trace width of 600 µm, gap between traces of 600 µm. The antenna is enclosed in a rectangle are with height of 48 mm and width of 78 mm (total area of 37.44 cm^2^). As reader, we fabricated a coil inductor milled in Flame-Retarded class 4 (FR-4) copper clad laminate rigid substrate from Cirqoid (Latvia) with a metallization layer of 35 µm copper using a prototyping machine from Cirqoid (Latvia). A surface mount device (SMD) capacitor was soldered to resonate at the desired frequency. The coil antenna was screen printed using a 100 Nylon threads per centimeter (T/cm) mesh. The antennas consisted of one deposited layer of silver paste with a manual screen printing machine (FLAT-DX 100 from Siebdruck-Versand, Magdeburg, Germany). The layer was dried at 120 °C for 5 min. The inner and outer ends of the coil were connected through a small “bridge” fabricated on polyimide by inkjet printing of silver and glued with the adhesive epoxy EPO-TEK H20E (Epoxy Technology, Inc., Billerica, MA, USA).

### 2.3. Characterization

The AC electrical characterization of the designed sensor tags was carried out by measuring magnitude and phase of the impedance, using the four-wire measurement technique with a precision Impedance Analyser E4294A and an impedance probe kit (42941A) (Agilent Tech., Santa Clara, CA, USA). In all measurements, the excitation voltage was set to V_DC_ = 0 and V_AC_ = 500 mV in the frequency range from 5 MHz to 30 MHz. We selected this range in order to cover the HF band (3–30 MHz) and we recorded 802 frequency points to be able to detect shifts on the resonance frequency due to variations in the ambient conditions.

The wireless sensor ware placed in a climatic chamber VCL 4006 (Vötsch Industrietechnik GmbH, Balingen, Germany) to characterize their responses toward humidity and temperature. The moisture content varied from 20%RH to 80%RH in steps of 20%RH every 30 min, whereas the temperature changed from 15 °C to 55 °C in steps of 10 °C every 30 min. We also introduced two commercial sensors close to the sensor tags to monitor the temperature and humidity: a digital thermometer RS Series A1 (RS Amidata S.A., Madrid, Spain) and an analogue humidity sensor HIH4000 (Honeywell International Inc., Chicago, Illinois, IL, USA).

**Figure 1:**
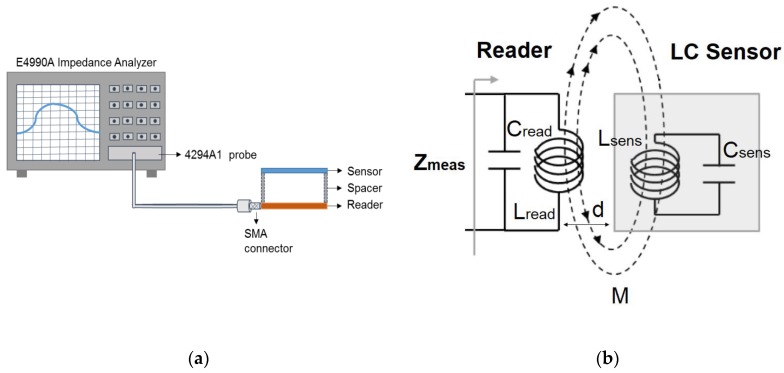
(**a**) Schematics of the set-up used for characterization; (**b**) Model circuit of the studied system.

The distance between the reader and the sensors was defined with a rectangular spacer manufactured with a 3D printer (EntresD UP Plus2, Germany) made of acrylonitrile butadiene styrene (ABS) with a perimeter of 85 mm × 53 mm. The height of this custom frame was checked with a digital calliper (DIN 862) with a resolution of 0.01 mm. The set-up is illustrated in Figure 1. Coils of sensor and reader were aligned during measurements in order to maximize the coupling factor.

## 3. Results and Discussion

### 3.1. Reader Characterization

**Figure 2:**
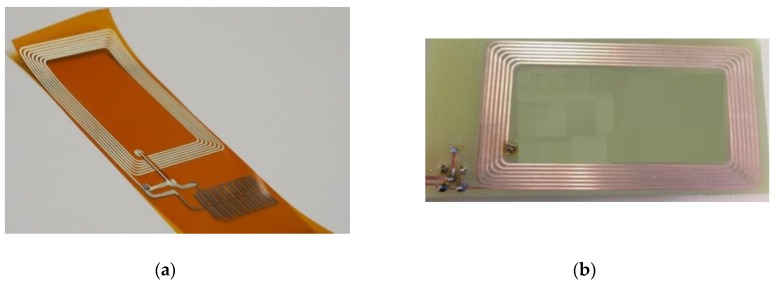
(**a**) Wireless sensor and (**b**) reader.

The prototypes employed as reader and as wireless sensor are presented in Figure 2. Both circuits consist of a coil inductor and a capacitor. In the reader, only one SMD capacitor is needed to tune the resonance frequency to the band of interest (HF); while in the case of wireless sensor, an array of capacitive structures is defined not only to tune the resonance frequency but to maximize the change in frequency because of RH variations.

**Figure 3:**
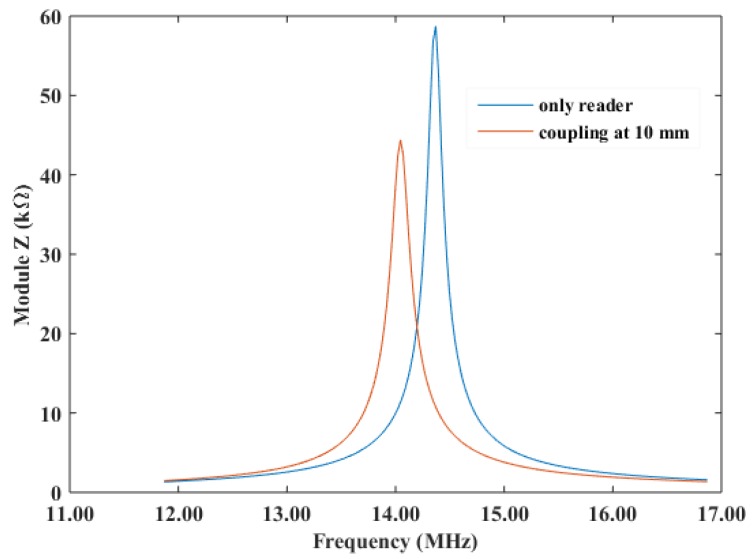
Module the reader and coupling between reader and wireless sensor at resonance condition.

The first study carried out was the frequency response of the reader antenna. The reader inductance value (L) is 5.99 µH, its quality factor (Q) is 59.9, and its self-resonance frequency (SRF) is 29.05 MHz. The inter-turn capacitance is 6.6 pF. An SMD capacitor of 18 pF was soldered in parallel leading to a resonance frequency of 14.52 MHz. In the case of the screen printed inductor, its values are L = 6.68 µH, Q = 2.08, and SRF = 30.05 MHz. The total value of the array of capacitances (12 out of 16 replicas) is 18.3 pF, thus no SMD was needed in this latter case. Figure 3 presents the module of the impedance of the reader in two different situations: when there is no tag in its surroundings and when a tag is located at 10 mm distance. In the former case (without any tag in its vicinity), the resonance frequency of the reader is at about 14.5 MHz, whereas it decreases approximately 0.5 MHz in the latter case. As stated in Equation 1, this reduction in the resonance frequency is related to the effect of the coupled sensor tag: the measured input impedance changes when this tag is in its surroundings [17], resulting in a shift on frequency as observed in Figure 3.

It should be noted that this relationship depends on the inductive coupling, and consequently, on the distance between the two elements. Thus, all the characterization must be done at a known distance. In the following sections, we will examine the response to RH and temperature for three different distances. It is important to clarify that in a real environment not only the reader antenna is important to define the whole system behavior, but also the full electronic design of the reader. One solution is to use a reader capable of detecting the resonance frequency of the wireless sensor [28,29].

### 3.2. Humidity Behavior

In a previous work, we already demonstrated that the impedance of the coil inductor fabricated on the same substrate remains virtually unaltered with respect to RH (less than 0.02% variation in frequency in the analyzed RH range), proving that the dominant element for modifying the resonance frequency in this kind of LC structure is the capacitive one: the array of IDE capacitors connected in parallel to obtain the desired global capacitance and sensitivity [20]. 

**Figure 4:**
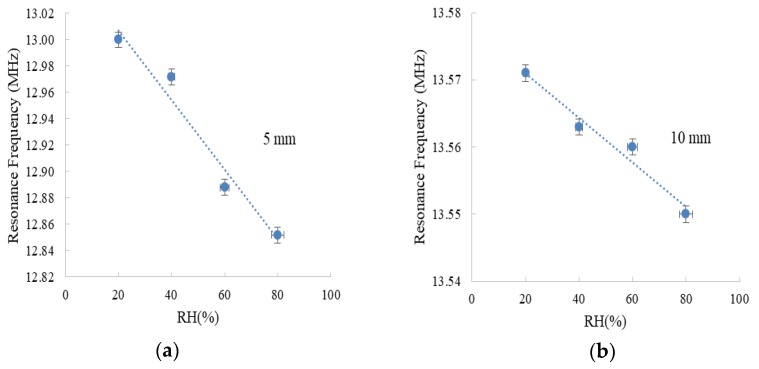

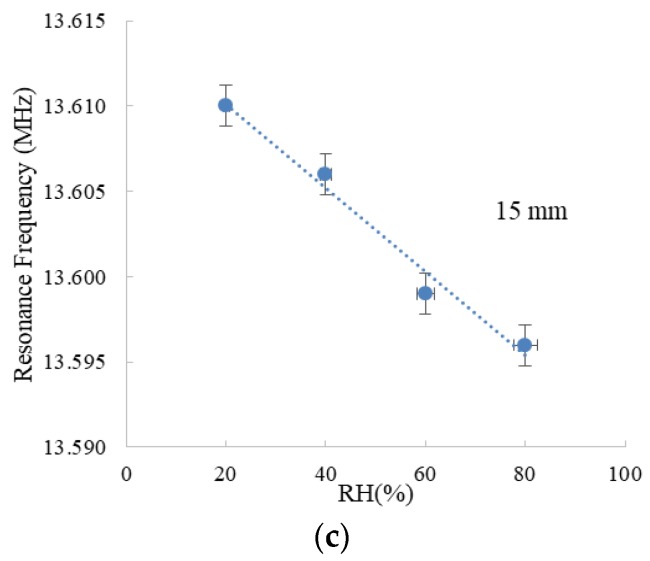
Resonance frequency of the reader while coupling the wireless sensor vs. RH level at 30 °C at a distance of (**a**) 5 mm; (**b**) 10 mm; (**c**) 15 mm. Error bars are calculated as the standard deviation of three measurement cycles of two different sensor tags.

Here, we characterized the wireless sensor at three different distances between reader and sensor tag. In particular, we located the wireless device at 5, 10, and 15 mm from the reader and measured the impedance seen by the reader (Figure 4). As the capacitance changes directly proportional to the moisture content, the resonance frequency decreases with the increase in RH. This response can be appreciated in the three studied scenarios and, at the same time, the influence of the distance between the two elements can be clearly observed: The variation of the resonance frequency with RH is much more perceptible at the shorter studied distance (5 mm). In particular, the resonance frequency shifted about 150 kHz at 5 mm, while this shift is one order of magnitude lower when we double the distance (10 mm). This substantial reduction of the impedance response with frequency emphasizes the important role of the distance in wireless chipless sensors: the characterization of the system can be completely different, from accuracy to detection limit. To handle this issue, we can follow different strategies. The simplest one is to use a fixed distance between the reader and the wireless sensor, but this limits the use of the system and a mechanism to control the reading distance should be included. Another solution is to include a reference tag [30,31] whose response does not change with respect to the parameter of interest (in our example, RH), so that the distance between reader and tag can always be inferred, and the calibration curve would be always known (including the distance d as part of this calibration curve).

Table 1 summarizes the variation in the resonance frequency (Res.freq) together with the calculated sensitivity within the analyzed RH range for the analyzed distances (dist.). The sensitivity to RH (S_RH_) is defined as follows:

(2) $$S_{\mathrm{RH}}\left(\mathrm{RH},\;\mathrm{dist}.\right)\equiv \frac{\partial \mathrm{Res}.{\mathrm{freq}}_{T=\mathrm{cte}}\;\left(\mathrm{RH},\;\mathrm{dist}.\right)}{\partial \;\mathrm{RH}}$$

For 5 mm separation, we can discriminate RH values from 20 to 80%RH in the range of hundreds of kHz, whereas the range decreased to tens of kHz at 10 mm distance and only a few kHz at 15 mm. Comparing the changes in the resonance frequency obtained when we measured at the reader side with the one presented in a previous paper [20] when we measured the variation in the sensor tag, the variation in the resonance frequency reduced about 500 kHz when we place the wireless sensor at 5 mm of the reader (and perform the measurement in the reader) instead of characterizing directly the response of the LC circuit. This reduction of about 70% of the shift in the resonance frequency highlights the importance of characterizing any wireless sensor system at the reader side.

Notice that the variation in the resonance frequency at each measure distance is caused by the change in alignment between both antennas.

This degradation in sensitivity can be attributed to the decay of the coupling coefficient (*k*) with distance, as explained in Section 2.1 [17]. The coefficient has an strong decay with distance, ($\frac{1}{d^{3}})$ [32] causing the reduction in the shift in frequency of reader’s SRF, as stated in Equation 1. Therefore, the shorter the distance between reader and sensor tags, the better the magnetic coupling and, thus, the higher the sensitivity of the wireless sensor system. 

### 3.3. Temperature Influence

It must be noted that we already proved that the variation passive structures with respect to temperature can be negligible [20], but we have studied how temperature affects two different RH levels, varying the distance between the elements of our systems. Table 2 summarizes the results of this study. In comparison with RH dependence, there is virtually no shift in the resonance frequency with temperature at the lowest tried distance (0.4 × 10^3^ Hz/°C at 50%RH), but it can be noticed that the thermal dependence increases at higher levels of RH (1.5 × 10^3^ Hz/°C at 70%RH). This interdependence is less important when the separation between the reader and the wireless chipless is bigger. Therefore, it is important, not only to control the distance between the two elements, but also to know the influence of the interfering parameters to avoid or reduce the uncertainty of the wireless chipless system.

The thermal dependence (S_T_) has been calculated using Equation (7)

(3) $$\;S_{T}\left(\mathrm{dist}.\right)\equiv \;\frac{\partial \mathrm{Res}.{\mathrm{freq}}_{\mathrm{RH}=\mathrm{cte}}\;\left(T\right)}{\partial \;T}\;$$

Table 3 summarizes the main contributions to wireless humidity sensors, pointing out their main features. It can be noticed that in all these cases, except for the current work, the influence of the distance to the reader antenna has not been considered. In our work, we prove the importance of the design of any wireless sensor together with the reader and operating conditions to optimize their performance. It can be seen that the response of the system [20] that previously showed a shift of 700 kHz between 20 and 90%RH when measuring directly among its terminals is drastically reduced, (about 5 times smaller) when measuring with a reader located a 1 cm from the sensor tag. Other works have taken care of analyzing the effect of the distance in similar scenarios but the sensor element was done on FR-4 substrate and no printing technique was employed for its fabrication [33,34,35]. They described a measurement method for capacitive sensors and demonstrated its application for humidity monitoring where the distance between the reader and the sensing tag can be compensated by measuring the impedance at different frequencies.

It is worth of mention that very little attention is paid to the influence of temperature in such LC sensors for RH monitoring, although it is well-known that the thermal drift is one of the most interfering factors when performing RH measurements. 

## 4. Conclusions

The characterization of a wireless chipless system is presented in this work, centering the study in the reader side. These kinds of systems are normally presented by describing only the frequency response of the sensor element and its variation towards the magnitude of interest but it is not analyze the real response of the system where the reader needs to read the sensor information and both elements are not directly connected. This is our motivation to describe the coupling characteristic of the chipless wireless sensor system at the reader side, as well as the influence of the distance between reader and wireless sensor in the impedance response of the reader. In particular, we have designed and fabricated a wireless sensor based on a LC circuit where the sensing element is the capacitive array printed on a substrate whose electrical permittivity is humidity dependent. This sensor tag have been located at different distances from the reader manufactured in FR-4 technology and the impedance seen by the reader while varying the moisture content have been studied. We have shown the importance of the distance between the devices for the proper and ubiquitous use of the system. In particular, a sensitivity of −2.6 kHz/%RH is observed at 5 mm distance while this value decreases more than 10 times when the separation between the reader and the wireless sensor is doubled (10 mm). This paper opens a new approach to perform the characterization of wireless chipless sensors in a more efficient way, closer to real environments. We suggest two strategies to implement this kind of system in the Internet of Things (IoT) paradigm: to fix the distance between the two elements or to add a reference element, whose response is invariant to the parameter of interest (in our case humidity).

## Figures and Tables

**Table 1:** Resonance frequency of the reader while coupling the wireless sensor at the minimum (20%) and maximum (80%) RH level tested for different distances.

Distance (mm)	Freq. Change (kHz)	Sensitivity (Hz/%RH)
10	148	−2.60 × 10^3^
15	21	−0.30 × 10^3^
20	14	−0.20 × 10^3^

**Table 2:** Resonance frequency of the reader while coupling the wireless sensor at the minimum (20 °C) and maximum (50 °C) temperature tested for different distances.

Distance (mm)	RH (%)	Freq. Change (kHz)
10	50	18 kHz
15	50	8 kHz
10	70	30 kHz
15	70	24 kHz

**Table 3:** Comparison among LC type sensor for humidity monitoring. * Distance between reader and tag antennas. Temp. stands for Temperature.

Reference	Fabrication Technology	Materials	Area (cm^2^)	Sensitivity (kHz/%RH)	Range RH (%)	Distance * (mm)	Temp. (°C)
Deen et al. 2014 [36]	Plasma enhanced Chemical Vapour Deposition	Graphene, Cu	<1	5.7	1–97	--	23
Zang et al. 2014 [37]	Spin-coating, patterning	Polyimide, Al	<1	65	10–95	0	25
Zang et al. 2015 [9]	CMOS technology and standard PCB process	GO, Cu	<1	−18.75	15–95	--	25
Feng et al. 2015 [38]	Inkjet printing	Paper, polyimide, PET	8	370	20–90	--	25
Wang et al. 2012 [21]	Dry-phase milling process, screen printing	Polyelectrolyte, Ag, carbon	100	−1.06	30–90	0	23
Fernandez-Salmeron et al. 2015 [20]	Screen printing, inkjet printing	Polyimide, Ag	40	−3.7	15–95	--	10–55
This work	Screen printing, inkjet printing	Polyimide, Ag	40	−2.60 to −0.20	20–80	10–20	10–55
